# Nursing Research, Dissemination of Knowledge and its Potential Contribution to the Practice

**DOI:** 10.17533/udea.iee.v40n3e01

**Published:** 2023-02-08

**Authors:** R. Mauricio Barría P

**Affiliations:** 1 . RN, MSc, Ph.D. Director of the Institute of Nursing, Faculty of Medicine, Universidad Austral de Chile. Chile. E-mail: rbarria@uach.cl Universidad Austral de Chile Faculty of Medicine Universidad Austral de Chile Chile rbarria@uach.cl

**Keywords:** nursing research, journal article, nursing, practical, evidence-based practice

Every day, nurses face dilemmas in clinical practice by having to make decisions about caring for patients, which can occur without up-to-date knowledge or sufficient resources. In previous reflections, I have exposed and commented on the need to move from research, particularly from research findings, to the practice in the different contexts of nursing work and of health sciences in general, exposing the gap that still remains between the generation of knowledge and its application in daily practice.(1,2) This gap, although in differential magnitude, is still present in the different scenarios in which nursing takes place, that is, in healthcare practice (hospital, outpatient, and community), as well as in teaching. 

Some ten years ago, Mitchell *et al*.,(3) presented a thematic analysis of the models to report on the development, transfer, and use of knowledge, identifying 47 models. These were grouped into thematic areas that accounted for the interaction among research, its adoption as knowledge, use, communication, and transfer. In addition, they highlighted that, despite progress in developing conceptual models, weak growth still prevails in aspects, like theoretical development related with Evidence-Based Practice, use of knowledge, and translational science.

If we focus on the care context, we should recognize that - as proposed - research contributes to the scientific rigor of the daily practice, allowing improvements by applying knowledge in favor of patient care; with such, clinical nurses could also be considered scientists.(4) Nonetheless, until now, it has been verified how the development of research fails to establish itself in clinical contexts and the nursing practice by clinicians themselves. Undoubtedly, research in teaching settings is greater as it is carried out by professors in academic contexts to understand problems and phenomena of practice in all its fields. In contrast, the real adoption of research as an effective function of nurses within their professional role still seems incipient. This, for example, is evident in reports that show that the participation of clinical nurses in scientific research and their perception on research capacity are suboptimal despite relatively high research training needs, so that they are not sufficiently prepared to conduct scientific research and rarely integrate scientific methodology in their clinical practice.(5) Moreover, a low level and quality of research conducted by hospital nurses has been detected, with poor participation in research groups.(6) 

Within this scenario, is that the use of available research to update and improve knowledge is of particular importance and, consequently, improve the clinical nursing practice, management, and teaching. However, we have known for a long time of the limitations to access and use evidence and even, in the first instance, to communicate research findings. 

Dissemination of knowledge derived from research is crucial, being able to share the results of different studies through oral communications in events and congresses and others, with greater reach and permanence in journal articles. That is why, given that we assume that the dissemination and use of knowledge in practice are necessary for professional growth, it needs to be disclosed after its publication in scientific journals, such as the setting provided by the journal *Investigación y Educación en Enfermería* that has permitted sharing progress and experiences in different areas of the discipline during nearly 40 years.(7)

This way, scientific publication is a critical point in how we renew knowledge in the discipline/profession and, thus, the scientific article becomes a fundamental aspect of the research process, allowing the closure of this path traced from an idea or problem and the response given to it thanks to the investigative process. From a shared perspective in the fields of research, the statement that "what is investigated and not written, or written and not published, is equivalent to not being investigated" becomes relevant.(8) 

Although it seems evident that an investigation is proposed knowing that it should be shared, a common problem limiting the dissemination of knowledge generated through research is the difficulty acknowledged by nurses in drafting a scientific article and for this reason, often, the effort to develop a research project and execute it is limited to it being shared in oral or poster presentations at scientific conferences, such as congresses or seminars. Notwithstanding, although this is a relevant step in communicating research findings, the impact is lower than publishing in a scientific journal. It is clear that the scope of publishing a work in academic journals allows sharing innovation experiences and contributing to learning within a field, which ultimately leads to improved patient care. However, it must be equally clear in pointing out that transforming a work from an abstract to an article to be submitted for review by a journal’s reviewers involves a greater challenge and requires developing specific skills.(9)

It is understandable that when limitations, difficulties, and barriers to conducting research, writing an article, and publishing it hinder the generation of new knowledge and its dissemination, clinical professionals must assume the responsibility and duty to update themselves based on the research available that constitutes the evidence that would allow improving care. In recent decades, initiatives have proliferated seeking to provide skills to improve the quality of nursing practice centered on the Evidence-Based Practice model. In this sense, an experience in the hospital context implemented a study plan based on this model, encompassing a number of guiding principles: shared governance, promotion, professional development, and EBP and, thereby, foster a spirit of inquiry to ensure resources to identify and use research findings in patient care.

Lastly, we must recognize the importance of strengthening the relationship and interaction between clinicians and academics as a way of establishing an alliance that promotes, firstly, collaborative research and continuous training for the search, analysis, interpretation, and application of available research. Thereby, proactivity to promote settings for permanent discussion will be the way research findings can effectively and efficiently impact the exercise and practice of nursing in all its areas. Otherwise, the gap will persist among practical nursing, graduate programs, and academia, making research infertile and that will continue to accumulate, failing to convey its main objective, which is competent, up-to-date, and ethical professional care for people and communities.

## References

[1] Barría RM (2017). The gap remains: The challenge of translating research into policies for the health care of people and communities. Invest. Educ. Enferm.

[2] Barría RM (2014). Implementing Evidence-Based Practice: A challenge for the nursing practice. Invest. Educ. Enferm.

[3] Mitchell SA, Fisher CA, Hastings CE, Silverman LB, Wallen GR (2010). A thematic analysis of theoretical models for translational science in nursing: mapping the field. Nurs. Outlook.

[4] Martínez Coronado DC, Camargo Sánchez A, Vargas Vargas RA, Camacho DM (2013). Investigación y ciencia en enfermería clínica: logros y desafíos. Actual. Enferm.

[5] Wu X, Wu X, Gao Y (2019). Research-training needs of clinical nurses: A nationwide study among tertiary hospitals in China. Int. J. Nurs. Sci.

[6] Silva-Vera ME, Bancalero-Herrera P, Ramos-Rodríguez JM, Morano-Alonso MC, Gallardo Cabrales S (2022). Investigación en cuidados: una aproximación a la realidad. Metas Enferm.

[7] Rodríguez-Gázquez MA, Chaparro-Hernández SJ, Rojas-Minota WM (2013). Scientific production of the journal Investigación y Educación en Enfermería during its 30 editing years. Invest. Educ. Enferm.

[8] Artiles Visbal L (1995). El artículo científico. Rev. Cubana Med. Gen. Integr.

[9] Tume LN, Vollam S, Trapani J (2021). How to convert your conference abstract into a paper for Nursing in Critical Care?. Nurs. Crit. Care.

[10] Burns HK, Noonan L, Jenkins DP, Bernardo LM (2017). Using Research Findings to Design an Evidence-Based Practice Curriculum. J. Contin. Educ. Nurs.

